# Nutritional Challenges of Active Sports Tourists: A Qualitative Study from the Runners’ Perspective

**DOI:** 10.3390/nu17142339

**Published:** 2025-07-17

**Authors:** Mateusz Rozmiarek

**Affiliations:** Department of Sports Tourism, Faculty of Physical Culture Sciences, Poznan University of Physical Education, 61-871 Poznan, Poland; rozmiarek@awf.poznan.pl

**Keywords:** sports tourism, sport, tourism, running, nutritional challenges, nutrition, dietary habits, half marathon, Poznan, Poland

## Abstract

**Background/Objectives:** Sports tourism, particularly international running events such as half marathons and marathons, has rapidly grown due to rising health consciousness and active lifestyles. Runners competing abroad face unique nutritional challenges that extend beyond physiological needs, including adaptation to local food cultures and psychosocial factors. This study aims to explore the nutritional difficulties encountered by international runners during competitions abroad, using participants of the Poznan Half Marathon 2025 as a case example. **Methods:** A qualitative research design was employed, involving semi-structured in-depth interviews with 12 international runners from the United Kingdom, Germany, and Ukraine. Participants had at least two years of experience competing internationally. **Results:** Four primary categories of nutritional challenges emerged: (1) quality and availability of food, (2) adaptation to local eating habits and physiological impacts, (3) hydration and access to appropriate fluids, and (4) logistical factors and the interactions between psychological stress, physical well-being, and nutritional choices. These factors influenced runners’ preparation, race-day performance, and recovery, highlighting the complexity of managing nutrition in unfamiliar environments. **Conclusions:** Nutritional challenges for international runners are multidimensional, requiring flexible and culturally sensitive nutritional strategies. Although these findings offer useful insights, they are based on a small, specific sample and should be generalized with caution. Further research is necessary to explore the broader applicability of the findings and their relevance to diverse athletic populations and contexts.

## 1. Introduction

Sports tourism has become one of the fastest-growing segments within the global tourism industry, reflecting broader societal trends toward health consciousness, active lifestyles, and experiential travel [1,2]. Over the past two decades, international participation in organized sporting events has significantly increased, with running events—particularly half marathons and marathons—gaining immense popularity among recreational athletes and amateur sports enthusiasts [3,4]. This rise is fueled by several factors, including the growing accessibility of international travel, the social appeal of participating in large-scale events, and the personal achievement associated with endurance sports [5]. Sport tourists, especially runners, often select destinations not solely for the competition itself but also for the cultural and environmental experiences that accompany travel [6,7,8,9]. Events such as the Berlin Marathon, the New York City Marathon, Poznan Marathon, or even the Poznan Half Marathon have transformed into global gatherings that merge athletic ambition with transnational mobility. As a result, sports tourism today is no longer a niche phenomenon but a culturally and economically significant form of tourism that blends physical activity, leisure, and international engagement [10,11,12]. Despite its scale, the unique needs and challenges faced by these mobile athletes, particularly in areas such as nutrition, health maintenance, and adaptation to new environments, remain underexplored in the academic literature. Thus far, this issue has been examined exclusively within the framework of sports volunteering, with existing investigations characterized by a relatively narrow analytical scope [13,14].

Among the diverse profiles of sport tourists, long-distance runners stand out as an active, particularly structured, and goal-oriented subgroup, often characterized by a high degree of planning and performance consciousness [15]. Unlike general tourists, runners participating in international events typically organize their travel around the race itself, prioritizing elements such as race-day logistics, proximity of accommodation to the starting line, and availability of pre-race meals aligned with their dietary routines. Their motivations are multifaceted—ranging from personal achievement and competition to cultural exploration and social interaction [16,17,18,19,20,21,22,23]—yet they often approach the journey with a clear physical and psychological preparation plan. Nutrition, hydration, rest, and routine become essential components of their experience, with many runners adhering to specific pre-race dietary strategies and post-race recovery protocols. This functional orientation toward the destination—where local cuisine and food environments are judged through the lens of performance readiness—positions runners as “conscious consumers” who seek a balance between athletic optimization, travel enjoyment, and pro-environmental behavior [24]. Consequently, the figure of the international runner embodies not only the active tourist, but also the informed and demanding participant whose expectations differ significantly from those of the average leisure traveler.

Nutrition plays a fundamental role in the preparation, performance, and recovery of middle- and long-distance runners, forming one of the most critical pillars of endurance sport [25,26]. For many athletes, especially those traveling abroad to participate in running events, dietary choices are not incidental but integral to their ability to complete the race and recover effectively. Pre-race nutrition often focuses on carbohydrate loading strategies designed to maximize glycogen storage in the muscles and liver, a practice shown to enhance endurance and delay the onset of fatigue [27]. During the run, maintaining hydration and managing energy levels through the intake of simple carbohydrates, electrolytes, or energy gels becomes vital in preventing performance decline, dehydration, or gastrointestinal distress [28]. Post-race nutrition, in turn, is aimed at accelerating recovery, repairing muscle tissue, replenishing glycogen, and reducing inflammation—typically through the timely consumption of carbohydrates, proteins, and fluids [29].

International runners participating in endurance events abroad often encounter a range of nutritional challenges that extend beyond physiological needs and enter the realm of cultural and environmental adaptation [30]. Traveling athletes must navigate unfamiliar food landscapes, limited access to preferred products, language barriers, and logistical restrictions such as hotel dining, early race hours, or venue-based meals. These practical constraints are further compounded by cultural differences in dietary norms, availability of staple foods, and varying definitions of what constitutes “healthy” or “performance-enhancing” nutrition [31,32].

The aim of this study is to understand the nutritional challenges faced by international runners participating in sporting events abroad, using participants of the Poznan Half Marathon as a case example. In this context, the research focuses on a single, central question: what logistical, psychological, cultural, and physiological nutritional difficulties do runners encounter when taking part in a sporting event outside their home country? To answer this question, qualitative interviews were conducted with twelve athletes from three different countries who traveled to Poland to compete in the race. Analysis of the participants’ responses allowed us to capture practical, cultural, and organizational aspects related to nutrition in the context of sports tourism, offering new insights into the importance of food availability, habits, and individual nutritional needs within the setting of an international competition.

## 2. Materials and Methods

The study employed a qualitative methodology centered on in-depth interviews (IDIs) [33]. IDIs are recognized as a highly effective technique for obtaining deep understanding of individuals’ perspectives and exploring diverse subjects [34]. By engaging with participants individually, rather than through group formats like focus groups, this approach facilitates more comprehensive data collection and encourages more open and detailed discussions, especially on sensitive or personal issues [35].

### 2.1. Sample Selection

This study included 12 international runners, comprising four women and eight men. Among them, four were from the United Kingdom, four from Germany, and four from Ukraine. All participants took part in the Poznan Half Marathon 2025 and reported having at least two years of experience traveling to various running events in different countries, meeting the criteria to be classified as sports tourists. The research focused on the challenges related to nutrition during stays abroad for running events and was part of a broader study examining various aspects and strategies associated with the sports tourism experience, as well as runners’ approaches to travel, nutrition, and physical activity abroad. All participants were of legal age. The sample was selected using purposive sampling, meaning individuals were chosen based on specific inclusion criteria: (1) permanent residence outside Poland and traveling specifically to participate in the Poznan Half Marathon 2025; (2) a minimum of two years’ experience traveling internationally for sports events. Participants who were underage or did not meet these criteria were excluded from the study.

### 2.2. Justification for Sample Size

The sample size was determined following qualitative research guidelines, which recommend that participant numbers are usually set based on reaching data saturation. Data saturation occurs when additional interviews no longer provide new, meaningful themes or insights that would enhance the understanding of the subject matter. In this study, saturation was reached after twelve interviews, indicating that the sample was sufficient to gather comprehensive and detailed information. This sample size is also consistent with recommendations found in qualitative research literature [36,37]. Despite being relatively small, the sample size was appropriate for the study’s exploratory goals and enabled the researcher to uncover nuanced, context-rich insights that would not be accessible through purely quantitative approaches.

### 2.3. Procedure

The study was conducted using semi-structured interviews, which enabled participants to openly share their perspectives through open-ended questions. All interviews were conducted in English. The interviews were carried out in April 2025, with each session lasting around one hour. All interviews were recorded with the explicit consent of the participants. The entire research process adhered to ethical scientific principles. Participants were thoroughly informed about the study’s objectives, its nature, and the voluntary nature of their participation. Informed consent was obtained from each individual for both their involvement and the recording of the interviews. Anonymity and confidentiality were strictly maintained, and the data were presented in a way that ensured participants could not be identified.

All interviews were conducted by the same researcher, which ensured consistency in interview style and helped build rapport with participants, facilitating richer and more detailed responses. The use of a semi-structured interview format with a consistent set of core questions across all interviews minimized variation in data collection, allowing comparability of responses while still providing flexibility for participants to express unique perspectives.

### 2.4. Data Analysis

The data collected from the interviews were analyzed using a structured four-step thematic analysis procedure: (1) transcribing the recordings and initially familiarizing with the material; (2) categorizing participants’ responses and identifying recurring themes; (3) grouping these categories into broader thematic clusters; and (4) interpreting the findings and drawing conclusions from the emergent themes. Each step adhered to established qualitative research standards to ensure the reliability and validity of the results.

The analysis was explicitly guided by Braun and Clarke’s [38] framework for thematic analysis, which provides a clear and systematic approach to identifying, analyzing, and reporting patterns within qualitative data. This framework enhances the credibility and replicability of the study by following a well-established analytical procedure.

To enhance the clarity and depth of the analysis, a multi-stage, iterative thematic analysis approach was employed. After transcribing the interviews, the transcripts were repeatedly reviewed to gain a comprehensive understanding of participants’ perspectives. Coding was conducted in two phases: (1) open coding to identify potentially important themes, followed by (2) axial coding to connect themes and highlight central patterns. This process was supported by analytical software such as NVivo (version 12), which facilitated a systematic and organized coding workflow.

The data analysis was conducted solely by the same researcher who performed the interviews. This approach ensured a deep contextual understanding of the data, as the analyst was intimately familiar with both the nuances of participants’ responses and the overall research objectives. While multiple analysts can reduce subjective bias, single-analyst interpretation allowed for consistent application of coding criteria and facilitated a cohesive and integrated interpretation of the data. All codes were then compared and consolidated to refine the thematic categories further. The findings were contextualized by comparing them with the limited existing literature in related fields, allowing for broader interpretation. Throughout the analysis, the researcher applied a careful and methodical coding strategy, frequently revisiting themes and cross-checking them against the original codes to minimize subjectivity and enhance the objectivity and consistency of the interpretations.

## 3. Results

The analysis of participants’ statements revealed four main areas of nutritional challenges faced by runners during competitions abroad. These categories emerged naturally from the data as clusters of frequently recurring themes and difficulties influencing athletes’ preparation and well-being. The four areas are (1) the quality and availability of food, (2) adaptation to local eating habits and their physiological impacts, (3) hydration and access to appropriate fluids, and (4) logistical factors along with the interactions between psychological stress, physical well-being, and nutritional choices. This classification captures distinct yet interconnected dimensions of runners’ experiences, providing a comprehensive understanding of the multifaceted challenges they face. By organizing the data in this way, the analysis addresses both practical and psychological aspects, facilitating clearer communication and targeted interpretation of the findings. Each of these topics is presented in the following section, illustrated with the original quotes from the study participants.

### 3.1. Quality and Availability of Food

One of the most frequently reported challenges regarding nutrition during participation in international running events was the limited availability of high-quality meals. Participants indicated that their dietary needs—especially within the context of a sports-oriented diet—were often overlooked in the standard food options available near race routes or accommodation facilities.

A respondent from the United Kingdom, who participated in races in Poland, explained, “It’s often difficult to find healthy food near the starting point, especially when I’m in Poland, where the cuisine is very greasy and based on heavy dishes. I’ve raced here several times, usually on Sundays. I really missed access to fresh vegetables or light meals, and that makes it very hard to maintain a proper diet”. This quote illustrates how local culinary preferences can clash with the nutritional needs of athletes. However, this issue was not limited to Poland—participants reported the lack of access to nutritious meals in other countries as well.

A participant from Ukraine described her experiences with lower-standard accommodations: “I usually stay in budget hotels or motels, where either there is no food provided, or the meals are monotonous and not very varied—and worst of all, they are rarely tailored to the needs of athletes”. Meanwhile, a respondent from Germany emphasized the dominance of highly processed foods: “Sometimes, there’s a real lack of good, balanced meals—for example, in many places, what’s mostly available is fast food or snacks loaded with sugar, which doesn’t help maintain performance. That means I have to rely on my own supplies, which can be difficult”. It is worth noting that these difficulties were both organizational and cultural in nature—participants often had to compromise between the local culinary offerings and their personal dietary preferences.

Challenges related to food quality and availability become even more complex for individuals who follow specialized diets or deal with food allergies. For many participants, the limited selection of suitable products requires additional planning and heightened caution. As one participant from Germany noted, “As a vegan, I struggle to find appropriate meals during races abroad. Local menus often revolve around meat or animal-based products, and vegan options are limited and don’t always provide the necessary energy”. Another respondent from the United Kingdom spoke about the difficulty of managing allergies: “I’m allergic to nuts, and unfortunately, in many countries they’re a very common ingredient in meals and snacks. I have to be extremely careful because even the smallest trace can trigger a reaction”. An additional concern was the lack of proper allergen labeling, as a German participant explained: “The absence of clear allergen information in foreign restaurants is a major challenge and source of stress for me. I often have to ask the staff, who don’t always know whether a dish is safe for me or not”.

Interestingly, none of the Ukrainian respondents raised issues related to special diets or food allergies. This may be due to the fact that Ukrainian cuisine and eating habits are culturally similar to Polish ones, which may ease the process of adaptation and reduce the sense of dietary discomfort during the events.

### 3.2. Local Food Culture and Bodily Adaptation

An important challenge that emerged in participants’ statements was the need to adapt to different eating habits and local food products, which often resulted in discomfort and, in some cases, a decline in well-being or physical performance. For many runners, the problem was not the food itself, but the lack of knowledge about its composition or preparation methods. A participant from Germany noted, “I don’t always know exactly what I’m eating because the ingredients and preparation methods in different countries differ from what I’m used to at home. Sometimes I come across completely unfamiliar spices that can affect how my body feels and responds”. Difficulties also arose with digesting exotic or heavy foods to which their bodies were not accustomed. As a participant from the United Kingdom observed, “Getting used to local dishes is problematic for me, as they’re often heavy or contain exotic ingredients that my stomach isn’t used to. This can negatively impact my performance during the race”. From the perspective of athletes who follow a specific pre-race nutrition plan, challenges were not only related to the food itself but also to the timing and portion sizes, which made it difficult to maintain a consistent routine. As another United Kingdom participant explained, “It’s hard for me to adjust to local eating customs, especially when I follow a strict nutritional plan before the race. I’ve noticed that in Poland, meals are eaten at different times or in different portion sizes, which complicates my diet”.

Environmental changes, such as differences in climate, water, food composition, or seasoning intensity, sometimes led to real physiological problems. A runner from Germany described, “During international races, I often experience stomach issues, which I suspect are caused by changes in food, water, or the overall environment. Even when I try to avoid risky dishes, my body sometimes reacts differently than usual”. In response to this challenge, some athletes deliberately avoided local cuisine in the days leading up to the race, although this came with added difficulty. As a participant from Ukraine explained, “I usually avoid unfamiliar foods for a few days before the race, but this approach really limits my choices, and sometimes it’s hard to find something that meets my needs”. A United Kingdom participant also emphasized the impact of local spices and unusual ingredients on their physical state: “My body often reacts poorly to local spices and dishes, which affects my form and endurance during the race. Even small dietary changes can make me feel sluggish or unwell”. These statements show that adapting to the local food culture is not merely a matter of taste or personal preference, but a tangible factor affecting an athlete’s physical readiness and race-day performance. Even minor deviations from routine can have significant effects on condition and overall well-being.

### 3.3. Hydration and Supplementation

Maintaining proper hydration and accessing familiar supplements posed a significant challenge for some respondents during their participation in international races. These issues most often stemmed from the limited availability of safe drinking water, the absence of known isotonic products, and difficulties in replenishing fluids during the race itself—especially under challenging weather conditions. A runner from Ukraine emphasized that he does not always trust local tap water, which forces him to seek alternative sources: “I can’t always trust tap water. On the other hand, buying bottled water can be expensive, and sometimes it’s hard to find, which makes it difficult for me to stay properly hydrated”. Similarly, a participant from the United Kingdom highlighted the difficulty of accessing specific functional beverages that are part of his supplementation routine: “The lack of availability of specialized isotonic drinks that I usually use to replenish electrolytes during races is a problem for me. I then have to look for alternatives”. Another Ukrainian respondent pointed to the lack of hydration infrastructure directly on the racecourse: “Sometimes there’s nowhere to refill fluids along the course, which is particularly problematic on hot days”.

It is worth noting that none of the German participants mentioned issues related to hydration or supplementation. This could suggest that they had better access to the necessary products or followed different preparation strategies—such as stocking up on water and drinks before traveling. It is also possible that this topic was simply not as relevant to them due to individual habits or prior experience.

### 3.4. Logistics and Psychophysical Factors

In addition to issues related to the availability or quality of food, participants also pointed to a range of non-dietary factors that significantly influence eating habits during race-related travel. These include both logistical concerns and psychophysical states—such as stress, fatigue, or the need for comfort.

Meal planning in unfamiliar settings, often without access to a private kitchen, requires considerable flexibility and organizational effort. A participant from the United Kingdom remarked, “Packing my own snacks and supplements becomes essential when I race abroad. While it gives me a sense of comfort, it definitely complicates travel and requires extra planning—especially when flights or transport options are limited”. A German runner also noted the difficulties caused by a lack of familiarity with the local food infrastructure: “Sometimes it’s very hard to plan meals when I don’t know the local shops, restaurants, or what they offer. As a result, I often end up eating whatever is available, which doesn’t always meet my needs”. Similarly, a Ukrainian respondent highlighted the challenges of self-preparation due to accommodation conditions: “Not having proper kitchen facilities at my accommodation is a major inconvenience—it prevents me from preparing a full meal. That forces me to eat out or buy ready-made meals, which are often less healthy”.

Beyond organizational difficulties, the effects of stress, tension, and fatigue emerged as significant challenges to proper nutrition. As one German participant shared, “The stress of competing often causes me to lose my appetite, and it becomes difficult to eat enough before a race. Even though I know how important nutrition is, sometimes my body just refuses to cooperate”. Travel-related fatigue, including from flights or adjusting to a new environment, also impacted participants’ appetite and meal quality. A United Kingdom runner noted, “Fatigue after flying often leaves me with no energy to eat. Unfortunately, that affects my performance later on”. Similar difficulties related to a tight schedule and lack of time for proper meals were pointed out by a participant from Ukraine: “The tight schedule of the race and my other tourist plans during the stay often leave me with little time to eat calmly and rest”.

Some runners also emphasized the need for psychological and sensory comfort. This often meant reaching for familiar products, even if they were not the healthiest options. For example, a participant from the United Kingdom admitted, “I definitely prefer to eat something familiar and tried-and-true, even if it’s not the healthiest option, because it gives me a sense of security and comfort. So I won’t lie—I often end up at popular fast-food chains”. For others, the main challenge was finding food that matched their individual taste preferences. As described by a German runner, “I can’t always find flavors I enjoy or that taste good to me. The lack of favorite foods often negatively affects my mood and energy level, which in turn impacts my performance”. Another respondent from the United Kingdom noted that choosing meals that were gentle on the stomach often led to intentionally avoiding local dishes: “There were many times I skipped meals because the local food was too heavy, too greasy, or too spicy for me. In such situations, it’s better to eat less, but choose something that sits well in the stomach”.

It is worth noting that none of the Ukrainian participants referred to taste preferences or difficulties adapting to local cuisine. This may be due to the fact that their culinary habits are relatively similar to those in Poland—both in terms of ingredients and preparation methods—which makes dietary adaptation less problematic.

## 4. Discussion

The conducted study highlighted the fundamental importance of food quality and availability for runners participating in international competitions. Difficulties in accessing fresh, easily digestible meals—as well as the prevalence of fast food or highly processed dishes—pose significant challenges that negatively impact athletes’ ability to maintain an optimal sports diet. In addition, participants emphasized that the monotony of meals in hotels and restaurants limits their nutritional options, which can lead to decreased energy levels and physical weakness at critical moments. This finding is consistent with previous research by Birkenhead and Slater [39] and Thurecht and Pelly [40], which showed that access to appropriate food is one of the key factors affecting athletic performance. Moreover, particular difficulties were reported by athletes following specialized diets, such as vegans or those with food allergies, who must remain especially vigilant and plan their meals with greater care. This indicates the need for better nutritional support and the inclusion of diverse dietary needs in the organization of international sporting events, for example, through food trucks that can offer more rational and athlete-appropriate food options, beyond the typical fast foods.

Another important aspect involves cultural differences and local dietary habits, which influence how well athletes’ bodies adapt during competitions abroad. Respondents pointed out that changes in ingredients, preparation techniques, and the use of unfamiliar spices often led to digestive issues and a general decline in well-being. The literature provides numerous examples showing that even short-term dietary changes can impact the gut microbiome and digestive efficiency [41]. Additionally, travel-related stress and pre-race anxiety, combined with fatigue from the journey, further worsen the situation—leading to a lack of appetite or digestive problems [42]. This complex interaction of external factors with the body underscores the need to integrate nutritional knowledge with stress management and recovery strategies in athletes’ preparation programs.

An interesting observation from the study is the relatively lower number of dietary or taste-related issues reported by Ukrainian participants. This may be attributed to strong cultural and culinary similarities between Poland and Ukraine, which ease dietary adaptation and minimize the stress associated with eating in a foreign country. This cultural proximity leads to a greater sense of comfort and fewer nutritional challenges, which aligns with literature discussing the impact of cultural factors on food perception and acceptance [43,44]. At the same time, participants from Germany and the United Kingdom more frequently reported difficulties related to unfamiliar flavors and preparation methods. This suggests that nutritional options should be individualized and take the athlete’s cultural context into account.

An important element of nutritional strategy during international competitions is the psychological comfort associated with eating. Many respondents reported choosing familiar and trusted products, even if they were not the healthiest options, because doing so gave them a sense of security and helped reduce pre-race stress. This behavior aligns with self-regulation theory, which posits that in high-stress situations, individuals tend to fall back on familiar behavioral patterns to maintain emotional stability [45]. This phenomenon highlights the need to address not only the physiological aspect of nutrition but also its psychological dimension, as this can contribute to improved performance and athlete satisfaction.

## 5. Study Limitations and Practical Implications

The study conducted had several limitations. It was qualitative in nature and based on participants’ subjective accounts, which entails certain limitations in terms of interpretation and generalization of the findings. The subjective nature of the responses may have influenced how participants perceived nutritional challenges and may have omitted unconscious factors affecting nutrition and well-being. Moreover, the research sample (12 interviews)—due to the qualitative methodology—was relatively small and limited to representatives from three countries (Germany, Ukraine, United Kingdom), which does not reflect the full diversity of experiences among runners from other regions or cultures. As a result, the findings cannot be fully representative of the entire population of international runners, as the absence of participants from non-European or more culturally distinct regions (e.g., Asia, Africa, or Latin America) limits the cultural depth of the study. Another limitation was the lack of quantitative methods, which would have made it possible to assess the scale and frequency of specific issues and allowed for statistical analysis of the relationships between variables. Without such data, it is difficult to determine which nutritional challenges are the most common or which groups of athletes are particularly affected. Additionally, the study did not account for seasonality or the specific characteristics of different running events, which can significantly impact the availability and quality of food and athletes’ nutritional strategies. Factors such as the time of year, the location of the event, or the length of the course can all create significant differences in athletes’ nutritional needs and experiences. Furthermore, since the data were collected exclusively from runners participating in the Poznan Half Marathon, location-specific factors—such as local cuisine, food availability, and infrastructure—may have influenced the results. This context-specificity may limit the applicability of the findings to other settings or events. Finally, a key limitation was the exclusion of a broader range of psychophysical and environmental variables that could influence nutrition and physical adaptation—such as stress levels, individual coping strategies for travel, or health status. Future research should aim to include larger and more diverse groups of athletes from various countries and cultural backgrounds, as well as integrate qualitative and quantitative approaches. This would allow for a more comprehensive understanding of nutritional challenges and support the development of more precise and effective recommendations for athletes, event organizers, and food providers.

It is essential to take into account the dietary and cultural diversity of participants by offering balanced and flexible food alternatives. Equally important is educating athletes on how to plan their nutrition during travel and how to cope with logistical and psychological challenges. Coaches and dietitians should collaborate to develop individualized nutritional strategies that consider the specifics of local foods, as well as the physiological and emotional needs of athletes. Finally, providing adequate kitchen facilities and the ability to transport personal food supplies can significantly enhance both comfort and dietary effectiveness during international competitions. In the context of sports tourism, such support becomes increasingly important, as participants must often navigate unfamiliar environments, altered dietary habits, and travel-related logistical challenges—underscoring the need to integrate nutrition into the broader planning of sports trips.

## 6. Conclusions

The conclusions drawn from the study indicate that nutritional challenges faced by runners during international competitions are multidimensional, encompassing issues related to food quality and availability, adaptation to local food culture, digestive problems, as well as logistical and psychophysical aspects. The diversity of diets and culinary habits, along with individual needs such as allergies or specialized diets, suggests a need for a flexible and informed approach to nutrition planning. However, given the small and specific sample of this study, caution is necessary when generalizing these findings beyond the participants involved. The results suggest that effective nutritional management—particularly in the context of traveling to international sports events—should consider cultural, physiological, and organizational aspects to potentially improve comfort, health, and athletic performance. The implementation of personalized nutritional strategies and improved collaboration between coaches, dietitians, food providers, and event organizers may help reduce nutritional problems and enhance participants’ overall satisfaction, although further research with larger and more diverse samples is needed to confirm these implications.

## Data Availability

The original contributions presented in the study are included in the article; further inquiries can be directed to the corresponding author.
